# Hybrid model for predicting microsatellite instability in colorectal cancer using hematoxylin & eosin-stained images and clinical features

**DOI:** 10.3389/fonc.2025.1580195

**Published:** 2025-06-23

**Authors:** Hangping Wei, Xiaowei Zhang, Zhen Zhou, Jianbin Xie, Weidong Han, Xiaofang Dong

**Affiliations:** ^1^ Department of Medical Oncology, Dongyang Hospital Affiliated to Wenzhou Medical University, Dongyang, Zhejiang, China; ^2^ Department of Pathology, Dongyang Hospital Affiliated to Wenzhou Medical University, Dongyang, Zhejiang, China; ^3^ Zhuoyue Honors College, Hangzhou Dianzi University, Hangzhou, Zhejiang, China; ^4^ Department of Respiratory Medicine, Dongyang Hospital Affiliated to Wenzhou Medical University, Dongyang, Zhejiang, China; ^5^ Department of Colorectal Medicine, Cancer Hospital of the University of Chinese Academy of Sciences (Zhejiang Cancer Hospital), Hangzhou, Zhejiang, China

**Keywords:** colorectal cancer, pathomics, prediction model, microsatellite instability, deep learning

## Abstract

**Background:**

Microsatellite instability (MSI) is a crucial molecular phenotype in colorectal cancer (CRC), which aids in determining treatment strategies and predicting prognosis. However, existing prediction methods have limitations and are not universally applicable to all patient populations. Consequently, we proposed a hybrid prediction model that integrates pathological and clinical features to predict MSI.

**Materials and methods:**

This study encompassed two patient cohorts: The Cancer Genome Atlas cohort (TCGA set, n = 559), which was divided into training and internal validation subsets at a ratio of 7:3, and the Dongyang CRC cohort (Dongyang set, n = 123), serving as an external testing cohort. Two deep learning approaches—semi-supervised and fully-supervised—were employed to extract features from pathological images. Subsequently, the pathomic signatures derived from these approaches were integrated with clinical features to develop a hybrid model. The hybrid model was assessed using an external validation cohort to determine the area under the curve (AUC). Furthermore, to investigate genes associated with MSI, we performed enrichment analysis and constructed a protein-protein interaction (PPI) network using mRNA sequencing data obtained from the TCGA database.

**Results:**

The fully-supervised pathological model demonstrated promising performance, achieving an AUC of 0.928 in the internal validation cohort, compared to the semi-supervised pathological model’s AUC of 0.786. In the external testing cohort, the model attained an AUC of 0.811. Subsequently, a hybrid model was established, which achieved an AUC of 0.949 in the validation cohort and a robust AUC of 0.862 in the test cohort. Additionally, a nomogram was developed to enhance its clinical applicability. Gene Ontology (GO) analysis identified differentially expressed genes (DEGs) related to MSI status, which were enriched in humoral immune response, among other pathways. Kyoto Encyclopedia of Genes and Genomes (KEGG) and Gene Set Enrichment Analysis (GSEA) revealed enrichment in pathways such as rheumatoid arthritis. A PPI network identified key hub genes, including IFNG and CD8A.

**Conclusion:**

The fully-supervised model consistently outperformed the semi-supervised model in predicting MSI. Furthermore, the hybrid model, which combines pathological and clinical features, demonstrated strong predictive ability.

## Introduction

CRC is the third most prevalent cancer worldwide and the second leading cause of cancer-related deaths (1, 2), with over 1.9 million new cases and one million deaths reported in 2020 (3). Microsatellites are short, repetitive DNA sequences, one to four base pairs in length, found throughout the genome. Their repetitive nature makes them prone to replication errors, which are typically corrected by mismatch repair (MMR) systems (4, 5). Mutations, deletions, or methylations affecting the MMR gene lead to the loss or impairment of its function, resulting in deficient mismatch repair (dMMR), a critical mechanism underlying MSI (6). MSI is a distinct mechanism that contributes to tumorigenesis in 10% of CRC cases and is a hallmark of hereditary Lynch syndrome-associated cancers (7). Identifying MSI is crucial for CRC management, as it significantly affects diagnosis, prognosis, and treatment planning (8). Patients with microsatellite instability-high (MSI-H) are a favorable group that can benefit substantially from immunotherapy for solid tumors (9). This highlights the critical role of MSI in advanced solid tumors.

Several diagnostic methods are commonly used to detect deficient dMMR or MSI in clinical settings, including immunohistochemistry (IHC) to identify MMR protein deficiencies and molecular tests such as polymerase chain reaction (PCR) or next-generation sequencing (NGS) (10). IHC testing requires optimal experimental conditions and skilled pathologists, along with access to tumor tissues (four proteins need to be tested), which can sometimes be insufficient. PCR or NGS testing requires specialized infrastructure that may not be universally available in hospitals, often leading to longer turnaround times or higher costs (11). Given these challenges, MSI testing is not universally applicable across all patient populations. Therefore, developing a universally accessible MSI testing method is imperative.

In routine clinical workflows, the diagnosis of CRC typically involves the histopathological evaluation of hematoxylin and eosin (H&E)-stained tissue slides, which can now be digitized into whole-slide images (WSIs) (12–14). WSIs offer comprehensive insights into the spatial organization of tumors, enabling examination at both low and high magnifications (9). Recent technological advancements, particularly in deep learning (DL), have revolutionized medical applications. DL-based algorithms are increasingly utilized in pathomics to enhance the accuracy and efficiency of disease diagnosis and prediction. These include tasks such as tumor diagnosis, subtyping, grading, staging, prognosis prediction, identification of pathological features, biomarkers, and genetic changes (15, 16). The integration of artificial intelligence (AI) with WSI analysis holds promise for improving diagnostic capabilities in CRC and other cancers, potentially overcoming some of the limitations associated with traditional testing methods such as IHC, PCR, and NGS.

**Figure 1:**
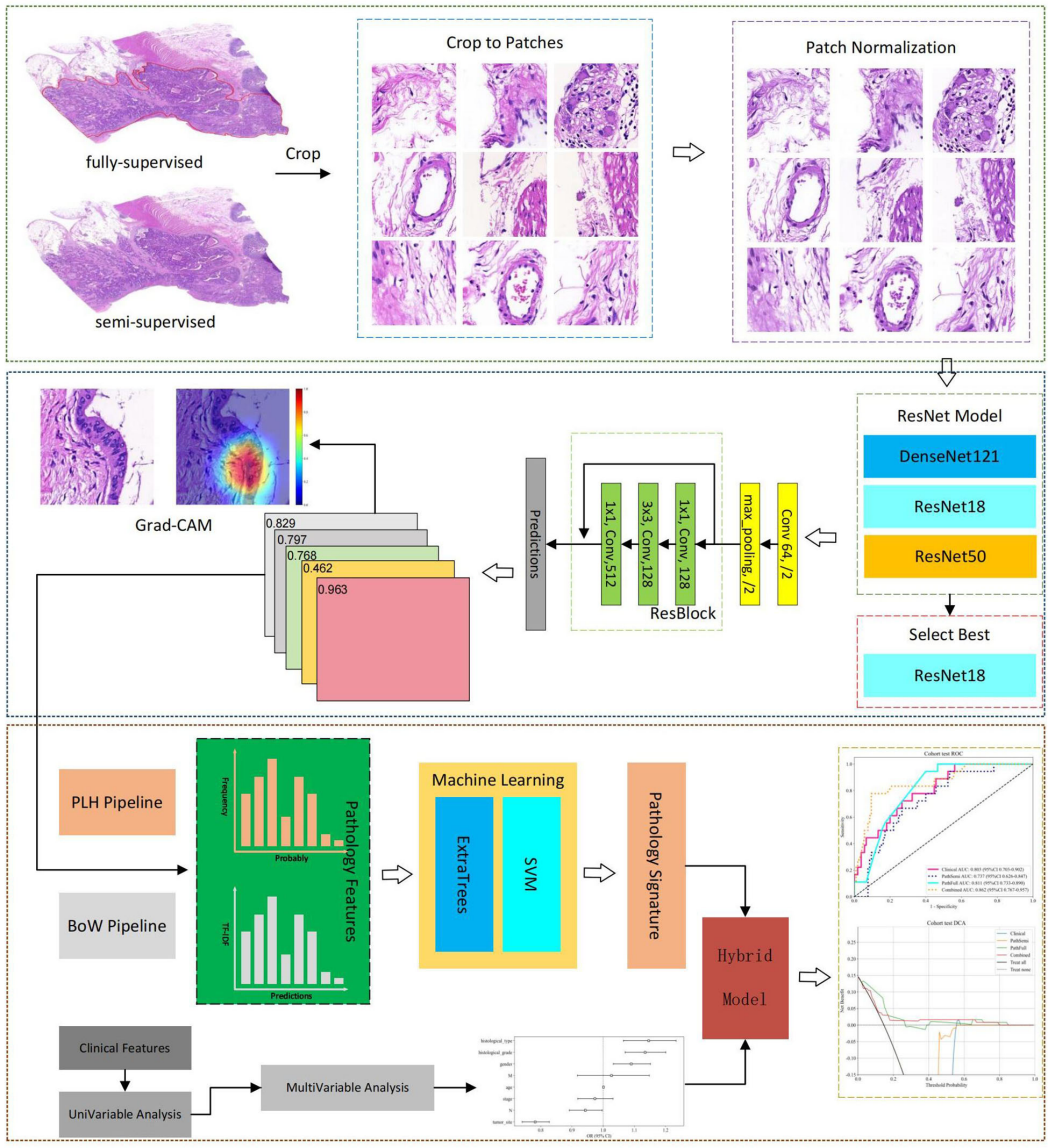
Workflow of the study for predicting MSI in CRC.

Although DL prediction of MSI has been extensively studied (9, 10, 17), previous research has primarily focused on predicting MSI using only pathological images, neglecting the integration of clinical patient characteristics. To address this, a hybrid prediction model that combines pathology slide data with clinical data was developed for predicting MSI in CRC. Both fully-supervised and semi-supervised learning methodologies were employed in DL pathological analysis. This comparative study evaluated the predictive advantages of both approaches, thereby enhancing the accuracy and robustness of the model and offering a scalable solution for predicting MSI status. The workflow of this study is illustrated in **Figure 1**.

## Materials and methods

### Patient cohort

For this study, WSIs from two large cohorts were collected, and each WSI was assigned an MSI label based on the MSI status of the patient. The first cohort (TCGA set, n = 559), was downloaded from the TCGA database (https://portal.gdc.cancer.gov/). Some samples were excluded from the analysis due to the absence of critical clinical data or incompleteness. Pathological images and clinical features, including gender, age, tumor site, histological grade, histological type, TNM stage, and vascular invasion, were also downloaded from the TCGA database and organized using loading packages such as string, maftools, and XML in R language software. The MSI status was determined based on the results from NGS analysis, as reported in the original research articles (18, 19). In this cohort, 483 and 76 cases were labeled as MSI-L/MSS (microsatellite instability-low/microsatellite stable) and MSI-H, respectively. The second cohort (Dongyang set), collected from Dongyang Hospital Affiliated to Wenzhou Medical University, comprised 123 formalin-fixed paraffin-embedded sections from patients diagnosed with CRC across all stages (between October 2021 and June 2023). H&E-stained images were digitized with PANNOROMIC MIDI II scanners (3DHISTECH, Hungary) using a 20× objective and saved as mrxs. format files. Through genetic testing of postoperative paraffin specimens, 105 and 18 cases were identified as MSI-L/MSS and MSI-H, respectively.

**Figure 2:**
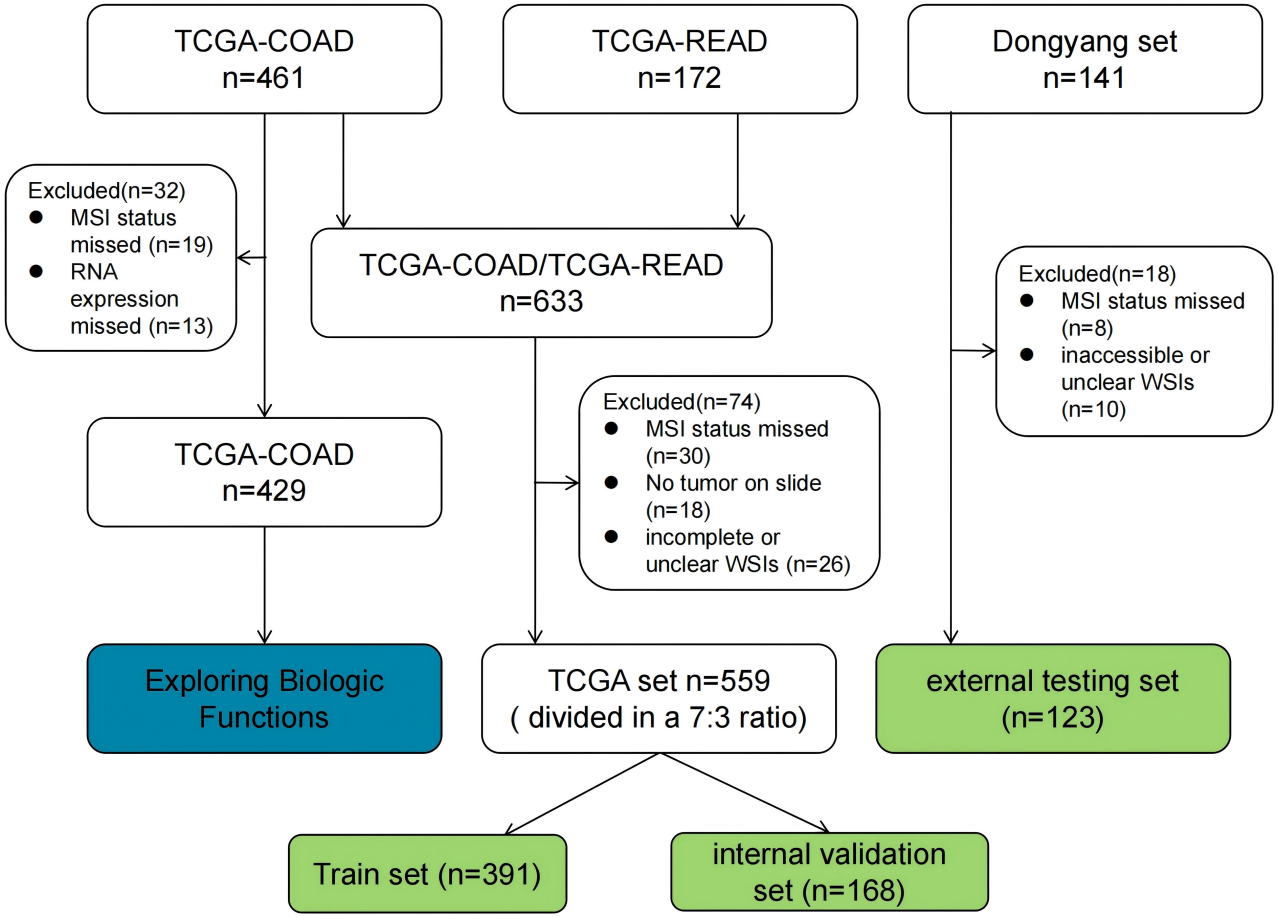
Flowchart of data collection and filtering for CRC patients from the two studies.

For model training, the TCGA dataset was divided into training and internal validation subsets in a 7:3 ratio. The training subset was used for hyperparameter tuning through cross-validation, while the internal validation subset was employed to evaluate the generalization performance. Additionally, an external testing dataset was incorporated to evaluate the generalizability of the model. A flowchart outlining the cohorts used in this study is shown in **Figure 2**.

### Regions of interest delineation and image preprocessing

WSIs were digitized at a 20 × magnification with a pixel resolution of approximately 0.5 μm/pixel. A fully-supervised DL approach involved manual annotation of cancer regions of interest (ROIs) by experienced pathologists using Qupath v0.5.1, with annotations subsequently reviewed for accuracy. In contrast, semi-supervised DL does not require manual annotation.

To enhance computational efficiency, algorithms based on Python were employed to automatically crop H&E-stained WSIs, each typically encompassing approximately 100,000 × 100,000 pixels, into smaller 512 × 512 pixel patches. Concurrently, patches with background areas exceeding 80% white, as well as those containing blurry artifacts or pen marks, were excluded from further analysis. All selected patches were normalized using the Macenko method to standardize color variations resulting from the staining procedures (20).

After normalization, these patches were used as inputs for the DL model. In cases where a slide produced more than 1,000 patches, a random selection of 1,000 patches was utilized for subsequent experiments to effectively manage computational resources. Throughout all experiments, only patient-level labels were applied, ensuring that all patches within the training sets inherited the labels of their respective parent patients. This method maintained consistency and integrity throughout the training process.

### Patch-level prediction

Our DL framework employs a dual prediction strategy: patch-level prediction and a multi-instance learning approach to integrate features from the WSI. During the training phase, the patches were assigned MSI labels based on the patient’s overall MSI status, which functioned as the training labels.

***Semi-supervised vs. fully-supervised:*** Two distinct patch selection methodologies were analyzed: a semi-supervised approach that utilizes the entire WSI and a supervised method that specifically targets tumor regions. Although the modeling processes for both methods are similar, the key difference lies in the selection of patches.

***Data augmentation***: To harmonize the intensity distribution across the RGB channels, Z-score normalization was applied to the images, preparing the data for input into the model. During the training phase, online data augmentation techniques, including random cropping and horizontal and vertical flipping, were employed. However, for testing patches, the processing was limited to normalization only.

***Model training:*** In this study, several renowned networks, including ResNet18, ResNet50, and DenseNet121, were analyzed to enhance the performance of traditional convolutional neural network (CNN)-based models at the patch level. Comparative evaluations were conducted on these models to identify the most effective algorithm tailored to our specific objectives. The details of the training process are outlined in **Supplementary 1A**.

### Patient level prediction

***Multi-Instance learning based feature fusion***: Upon training the DL model, the prediction phase began. During this phase, labels and corresponding probabilities were assigned to all patches. These patch probabilities were subsequently aggregated using a classifier to extract features at the WSI level. Two unique methodologies were developed to synthesize these patch probabilities, as detailed in **Supplementary 1B**.

1. ***Patch likelihood histogram pipeline***: This method employs a histogram to illustrate the distribution of patch likelihoods throughout a WSI. The histogram effectively captures the entire spectrum of likelihoods, providing a detailed depiction of the WSI.
2. ***Bag of words pipeline***: By integrating histogram- and vocabulary-based concepts, this method applies term frequency-inverse document frequency (TF-IDF) mapping to each patch. The resulting TF-IDF feature vector encapsulates the characteristics of the WSI.

The two pipelines facilitate the effective integration of patch-level predictions into comprehensive WSI-level features, making them suitable for advanced analyses such as metastasis prediction and survival analysis.

***Feature selection***: A total of 206 features were aggregated in this study using multi-instance learning through two distinct processes, each contributing 101 probability features and 2 predictive label features. To refine this feature set, a correlation-based selection method was applied, retaining only one feature from any pair with a Pearson’s correlation coefficient exceeding 0.9. Consequently, our feature set was reduced to two distinct features, which were subsequently used in the development of two machine-learning algorithms: SVM and ExtraTrees.

### Model building

***Pathology model***: Patch-level predictions, probability histograms, and TF-IDF features were synthesized in this study to construct detailed patient profiles. These comprehensive features served as the primary input for developing a specialized machine-learning algorithm tailored for MSI.

***Clinical model***: Mirroring the pathology model, a machine learning model for MSI analysis, which focuses on clinical features, was used. The model generated predictions that were particularly attuned to these clinical characteristics.

***Combined model***: To identify significant predictors, we conducted both univariate and multivariate analyses on these features. Features with a p-value of less than 0.05 from the multivariate analysis were integrated into the pathology model, resulting in a combined model. To enhance its clinical applicability and improve its interpretability and usability in clinical settings, this combined model was visualized using a nomogram.

***Metrics***: To evaluate the discriminative capabilities of all models in classifying the three types of pathologies, both macro- and micro-AUC metrics were used. These metrics provide a comprehensive assessment of the effectiveness of the algorithmic models in distinguishing between different pathology types.

### Exploring biologic functions

The mRNA sequencing data (TCGA-COAD, n = 429) was extracted from the TCGA database, and the MSI status of the relevant patients was identified from the original research article (18, 19). Differential analysis between MSS/MSI-L and MSI-H groups was conducted using the “limma” package, with a preset threshold of |log2FC| > 1 and a p-value < 0.05. The “clusterprofiler” package was utilized for GO and KEGG enrichment analysis on DEGs. Furthermore, GSEA on all genes was performed to visualize the primary activation pathways of the three enrichment analyses. To delve deeper into the molecular mechanisms, the PPI network of DEGs was constructed using Cytoscape software, and the CytohHubba plugin was employed to select the top 10 core proteins based on their degree values within the PPI network.

### Statistical analysis

The Shapiro–Wilk test was used to assess the normality of the clinical feature distribution within these cohorts, followed by t-tests or Chi-squared () tests, as appropriate, for a more in-depth analysis of the clinical features. The analysis was conducted using Python version 3.7.12, which incorporates a suite of specialized packages including Pandas 1.2.4 for data manipulation, NumPy 1.20.2 for numerical operations, PyTorch 1.8.0 for deep learning tasks, Onekey 3.1.3 for streamlined processing, OpenSlide 1.2.0 for handling whole slide images, SciPy 1.7.3 for scientific computing, Scikit-learn 1.0.2 for machine learning algorithms, and Slideflow 2.1.0 for pathology image analysis. All tests were two-sided, and p < 0.05 indicated statistical significance.

## Results

To optimize the model’s hyperparameters, a 5-fold cross-validation approach, in conjunction with the GridSearch algorithm, was applied to 70% of the dataset designated as the training set. After identifying the optimal hyperparameters, the entire training set was utilized to train the final model.

### Clinical features

**Figure 3:**
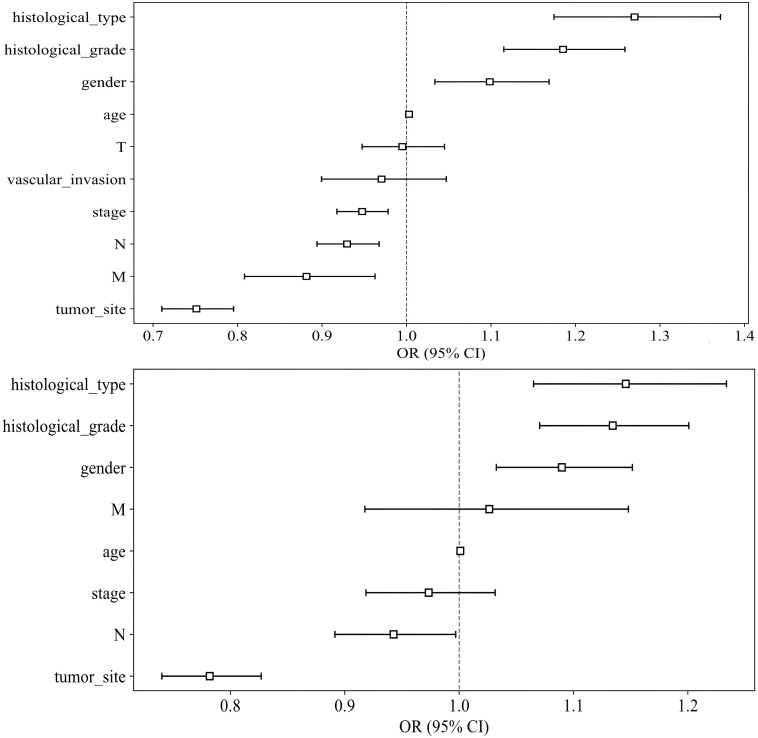
Odds Ratios of Clinical Features in Univariable Analyses (above) and Multivariable Analyses (below).

***Univariable and multivariable analyses***: To identify significant clinical predictors of MSI, we conducted a univariate analysis on all clinical features, calculating the odds ratio (OR) and associated p-value for each feature. Features such as age, gender, tumor site, histological grade, and type, as well as N, M, and TNM stage, were statistically significant (p < 0.05). Consequently, gender, tumor site, histological grade, and histological type were selected for inclusion in the combined model through multivariable analysis. The baseline characteristics of the patients in the two cohorts are presented in **Table 1**. Univariable and multivariable analyses of the clinical features for predicting MSI are presented in **Table 2**. Univariable and multivariable analyses of the OR for the clinical features are shown in **Figure 3**.

**Table 1:** Baseline clinical characteristics of patients in the two cohorts.

Feature_name	TCGA set (n=559)		Dongyang set(n=123)	Pvalue
	Training set(n=391)	Internal validation set(n=168)	External testing set(n=123)	
age	66.94 ± 12.70	65.03 ± 13.08	63.24 ± 13.58	0.118
gender				0.279
male	212(54.22)	82(48.81)	66(53.66)	
female	179(45.78)	86(51.19)	57(46.34)	
tumor_site				0.016
right	187(47.83)	61(36.31)	60(48.78)	
left	204(52.17)	107(63.69)	63(51.22)	
histological_grade				0.546
G1	19(4.86)	5(2.98)	4(3.25)	
G2	281(71.87)	126(75.00)	89(72.36)	
G3	91(23.27)	37(22.02)	30(24.39)	
histological_type				0.044
adenocarcinoma	321(82.10)	150(89.29)	101(82.11)	
special type adenocarcinoma	70(17.90)	18(10.71)	22(17.89)	
T				0.241
1	14(3.58)	5(2.98)	1(0.81)	
2	68(17.39)	23(13.69)	5(4.07)	
3	270(69.05)	114(67.86)	38(30.89)	
4	39(9.97)	26(15.48)	79(64.23)	
N				0.177
0	231(59.08)	85(50.60)	65(52.85)	
1	91(23.27)	48(28.57)	28(22.76)	
2	69(17.65)	35(20.83)	30(24.39)	
M				0.687
0	335(85.68)	141(83.93)	107(86.99)	
1	56(14.32)	27(16.07)	16(13.01)	
stage				0.243
I	74(18.93)	23(13.69)	4(3.25)	
II	150(38.36)	59(35.12)	61(49.59)	
III	111(28.39)	59(35.12)	42(34.15)	
IV	56(14.32)	27(16.07)	16(13.01)	
vascular_invasion				1
no	311(79.54)	133(79.17)	90(73.17)	
yes	80(20.46)	35(20.83)	33(26.83)	

**Table 2:** Univariate and multivariate analyses of clinical features for MSI prediction.

Feature name	Univariable Analysis				Multivariable Analysis			
	OR	OR lower 95%CI	OR upper 95%CI	p_value	OR	OR lower 95%CI	OR upper 95%CI	p_value
age	1.003	1.001	1.005	<0.05*	1.001	0.999	1.003	0.401
gender	1.099	1.034	1.169	<0.05*	1.090	1.033	1.151	<0.05*
tumor_site	0.751	0.710	0.795	<0.05*	0.782	0.740	0.827	<0.05*
histological_grade	1.185	1.115	1.259	<0.05*	1.134	1.070	1.201	<0.05*
histological_type	1.270	1.175	1.372	<0.05*	1.146	1.065	1.234	<0.05*
T	0.995	0.947	1.045	0.859				
N	0.930	0.894	0.968	<0.05*	0.943	0.891	0.997	0.083
M	0.882	0.808	0.963	<0.05*	1.027	0.918	1.148	0.698
stage	0.947	0.918	0.978	<0.05*	0.973	0.919	1.031	0.438
vascular_invasion	0.971	0.899	1.047	0.521				

* Indicates P less than 0.05, which is statistically significant.

### Patch level prediction

#### Patch level efficiency

Here, we illustrate the process using a fully supervised approach as an example. During the validation phase, the ResNet18 model demonstrated a moderate ability to differentiate between classes, achieving an AUC of 0.763. Additionally, ResNet18 outperformed all other models in the test cohort. Although its AUC was lower during the training phase (AUC = 0.945), ResNet18 was selected for further analysis due to its relative robustness and effectiveness in the validation phase, outperforming both ResNet50 and DenseNet121. The detailed performance metrics, including accuracy and AUC, are summarized in **Table 3**.

**Table 3:** Metrics for training, validation, and test cohorts in MSI prediction at the patch level using fully-supervised methods.

ModelName	Cohort	Acc	AUC	95% CI	Sensitivity	Specificity	PPV	NPV
resnet18	train	0.869	0.945	0.9447-0.9455	0.854	0.872	0.623	0.960
resnet18	val	0.702	0.763	0.7616-0.7652	0.691	0.703	0.232	0.946
resnet18	test	0.596	0.646	0.6447-0.6479	0.668	0.580	0.259	0.888
resnet50	train	0.902	0.970	0.9693-0.9698	0.898	0.903	0.697	0.973
resnet50	val	0.633	0.717	0.7150-0.7189	0.718	0.622	0.198	0.944
resnet50	test	0.590	0.643	0.6416-0.6449	0.633	0.580	0.249	0.878
densenet121	train	0.895	0.963	0.9629-0.9635	0.882	0.898	0.681	0.968
densenet121	val	0.657	0.725	0.7226-0.7266	0.664	0.656	0.201	0.938
densenet121	test	0.694	0.638	0.6366-0.6398	0.487	0.740	0.291	0.868

A detailed description of the semi-supervised methods is listed in **Supplementary 1C**.

### Grad-CAM visualization

**Figure 4:**
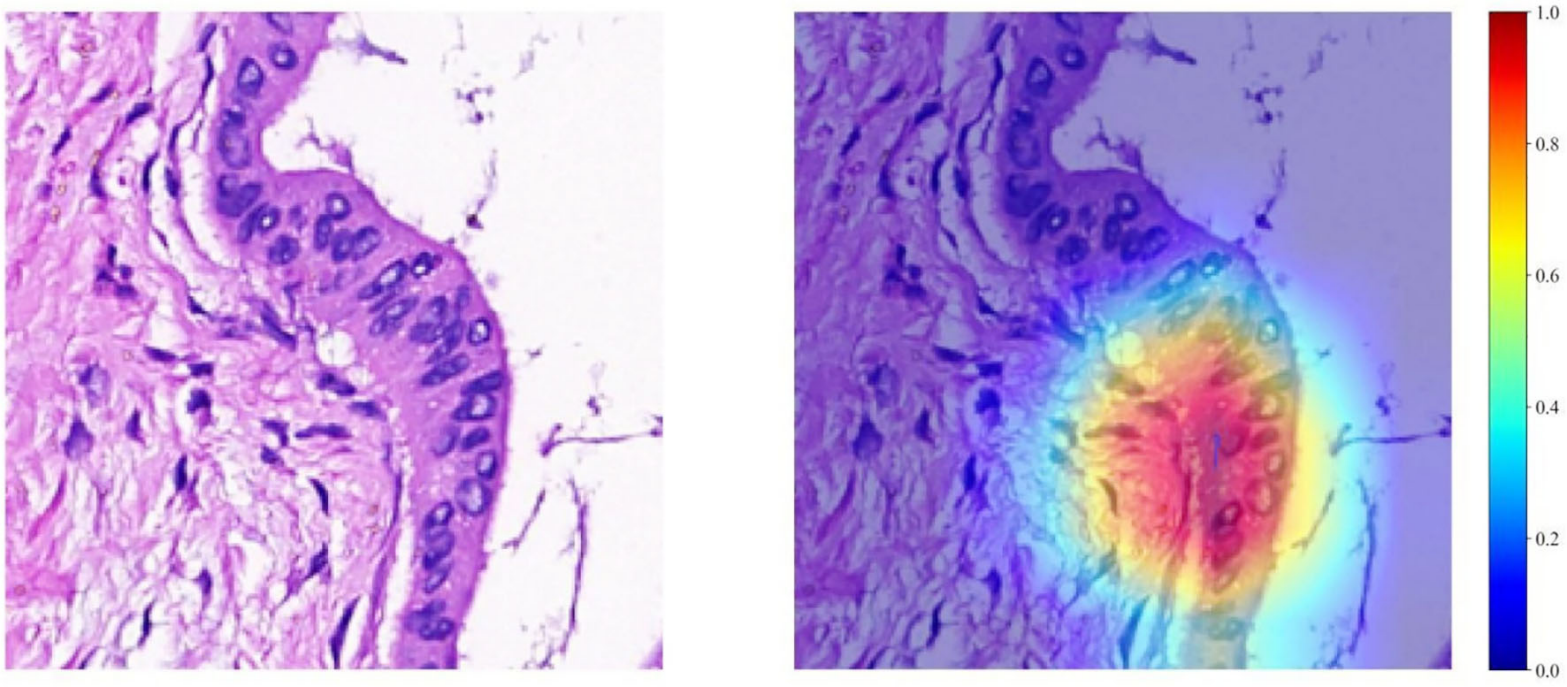
Visualizations for a single patient, comprising a tile image and its corresponding heat map. In the heat maps, regions highlighted in red signify areas of higher weight, as indicated by the color bar on the right side of the figure.

The Grad-CAM method allows for the generation of activation maps without altering the existing model architecture or necessitating additional training. As depicted in **Figure 4**, Grad-CAM is utilized to visualize the activations within the final convolutional layer, which is responsible for predicting the MSI. By making this layer transparent, Grad-CAM emphasizes the areas of the input image that are most significant in the model’s decision-making process. This technique provides valuable insights into the model’s reasoning behind its predictions, without requiring complex changes to the architecture or retraining.

### Patient level prediction

**Figure 5:**
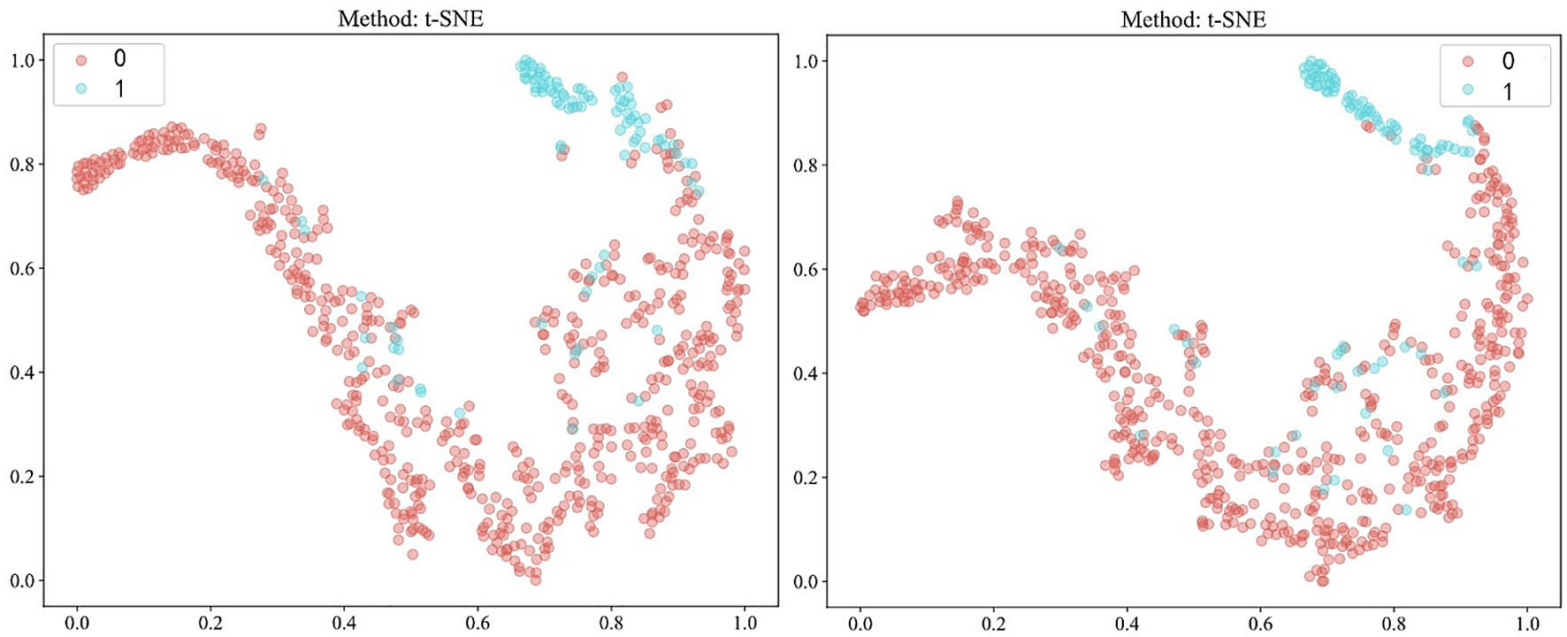
Visualization of patient-level features using t-distributed stochastic neighbor-embedding (t-SNE) after Pearson’s correlation analysis, comparing fully-supervised (left) with semi-supervised (right). 0: MSS/MSI-L; 1: MSI-H.

Our study incorporated data from 682 patients, each characterized by binary outcomes (0 or 1), with the objective of predicting these outcomes using features aggregated through multi-instance learning. A total of 206 features were compiled using this approach. To streamline this feature set, we applied a correlation-based selection technique, retaining only one feature from each pair with a Pearson correlation coefficient exceeding 0.9. The refined feature set was subsequently used in various machine-learning models for further analysis. To effectively visualize these features, we employed a t-distributed stochastic neighbor-embedding (t-SNE) algorithm. The results of the visualization process are depicted in **Figure 5**.

***Metrics:*** Here, we similarly illustrate the process using a fully supervised approach as an example. In the context of multi-instance learning, the ExtraTrees model demonstrated superior performance with an AUC of 0.928 in the validation cohort. This performance notably exceeded that of the SVM model, which had an AUC of 0.876. Although both the ExtraTrees and SVM models experienced a decline in their validation performance, the AUC remained relatively higher for ExtraTrees than for SVM (0.811 vs. 0.789) in the test cohort. The metrics for the training, validation, and test cohorts for predicting MSI using the fully-supervised pathomics model are presented in **Table 4**.

**Table 4:** Metrics for the training, validation, and test cohorts in MSI prediction using the fully-supervised pathomics model.

model_name	Cohort	Accuracy	AUC	95% CI	Sensitivity	Specificity	PPV	NPV
SVM	train	0.964	0.906	0.841 - 0.970	0.889	0.979	0.889	0.979
SVM	val	0.804	0.876	0.777 - 0.975	0.769	0.806	0.250	0.977
SVM	test	0.626	0.789	0.701 - 0.878	0.889	0.581	0.267	0.968
ExtraTrees	train	0.982	0.995	0.988 - 1.000	0.937	0.991	0.952	0.988
ExtraTrees	val	0.875	0.928	0.883 - 0.973	0.846	0.877	0.367	0.986
ExtraTrees	test	0.789	0.811	0.733 - 0.890	0.556	0.829	0.357	0.916

Information on semi-supervised methods is provided in **Supplementary 1D**.

**Figure 6:**
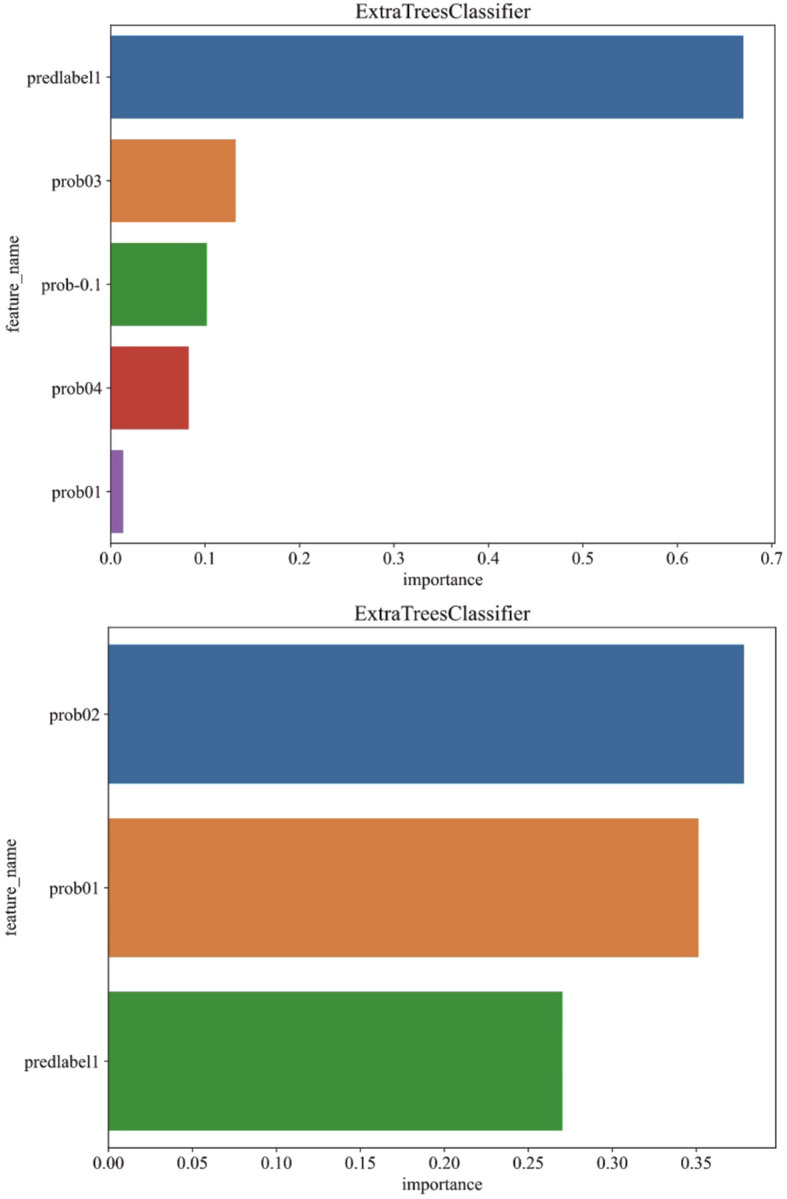
Analysis of feature importance in multi-instance learning under fully-supervised (above) and semi-supervised (below) approaches.

To further explain the pathological prediction model, we conducted an analysis of feature importance in multi-instance learning under both fully-supervised and semi-supervised approaches, as depicted in **Figure 6**.

### Signature comparison

In our analysis, the best-performing models were selected from the validation cohort for both the clinical and pathological frameworks. For the pathological model (fully-supervised), ExtraTrees was selected due to its superior performance, whereas, given its effectiveness, SVM was chosen for the pathological model (semi-supervised) and the clinical model. Additional details of the clinical model are provided in **Supplementary 1E**.

**Figure 7:**
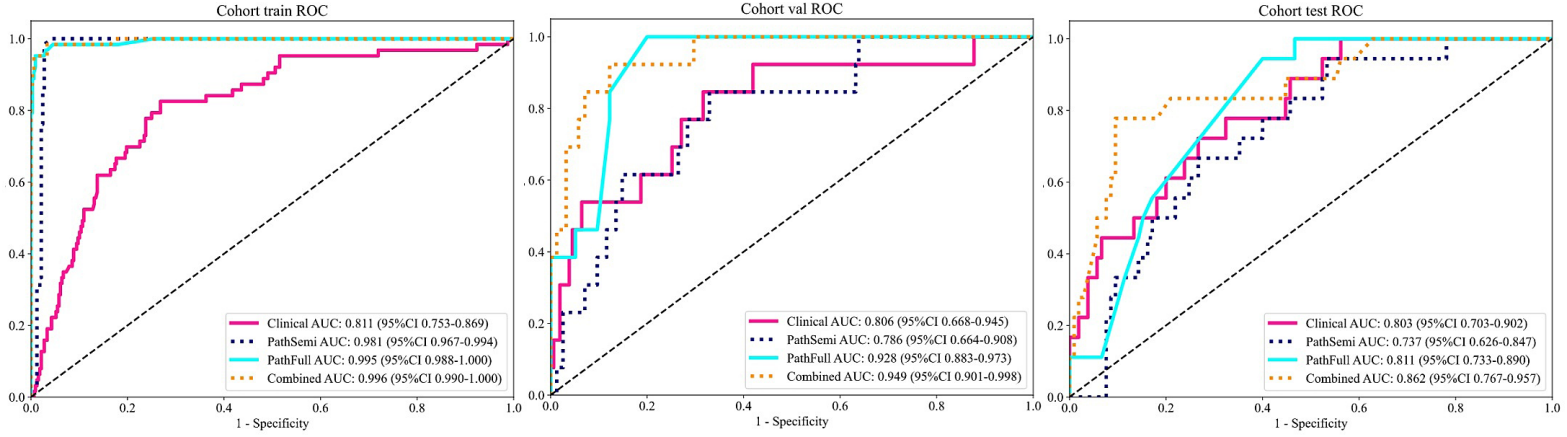
Variations in Area Under the Receiver Operating Characteristic Curve (AUROC) across all cohorts.

Furthermore, the combined model was included in this comparison to evaluate its performance relative to the individual clinical and pathological models. The “combined” model demonstrated superior performance across different cohorts, achieving the highest AUC of 0.996 in the training cohort and an AUC of 0.949 in the validation cohort, indicating exceptional predictive accuracy. Although there was a slight decrease in the test cohort, the “combined” model still maintained a robust AUC of 0.862, significantly outperforming the standalone “Clinical,” “PathSemi,” and “PathFull” models in the same setting (refer to **Table 5**, **Figure 7**).

**Table 5:** Performance of MSI Prediction Using Individual and Combined Models.

Signature	Cohort	Accuracy	AUC	95% CI	Sensitivity	Specificity	PPV	NPV
Clinical	train	0.373	0.811	0.7534 - 0.8688	0.968	0.259	0.201	0.977
PathSemi	train	0.974	0.981	0.9670 - 0.9943	0.984	0.973	0.873	0.997
PathFull	train	0.974	0.995	0.9876 - 1.0000	0.952	0.979	0.896	0.991
Combined	train	0.987	0.996	0.9903 - 1.0000	0.952	0.994	0.968	0.991
Clinical	val	0.435	0.806	0.6679 - 0.9450	0.923	0.394	0.113	0.984
PathSemi	val	0.917	0.786	0.6642 - 0.9080	0.231	0.974	0.429	0.938
PathFull	val	0.905	0.928	0.8829 - 0.9732	0.385	0.948	0.385	0.948
Combined	val	0.940	0.949	0.9010 - 0.9977	0.385	0.987	0.714	0.950
Clinical	test	0.447	0.803	0.7028 - 0.9025	1.000	0.352	0.209	1.000
PathSemi	test	0.821	0.737	0.6263 - 0.8467	0.000	0.962	0.000	0.849
PathFull	test	0.862	0.811	0.7328 - 0.8900	0.111	0.990	0.667	0.867
Combined	test	0.870	0.862	0.7668 - 0.9575	0.111	1.000	1.000	0.868

The “combined” model has proven to be a comprehensive and reliable predictive tool. Its strong performance across training, validation, and test cohorts highlights its robustness for clinical use, offering greater generalizability and reliability than single-source models due to the integration of diverse data sources and learning strategies.

***Calibration curve***: The Hosmer–Lemeshow (HL) test statistic is a key metric for evaluating the calibration of predictive models, reflecting how well predicted probabilities align with actual outcomes. Typically, a higher HL test statistic indicates better calibration, meaning that the model’s predictions are more closely aligned with the observed results. In our analysis, the nomogram model demonstrated excellent calibration performance across all cohorts, with HL test statistics of 0.498, 0.425, and 0.193 for the training, validation, and test cohorts, respectively.

### Clinical use

**Figure 8:**
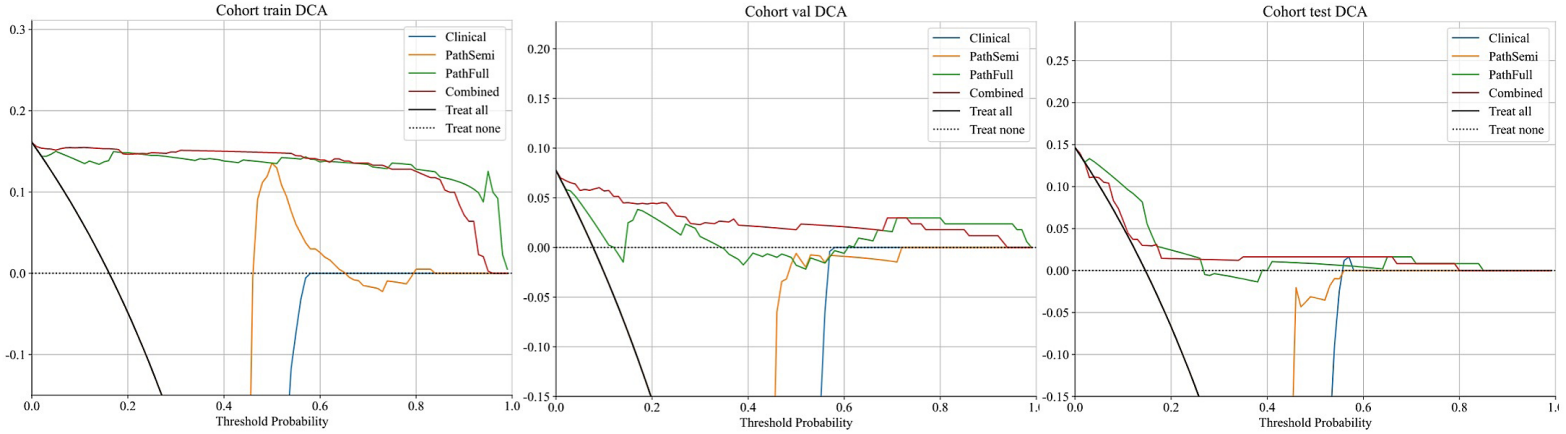
Decision curves for various signatures across all cohorts.

**Figure 9:**
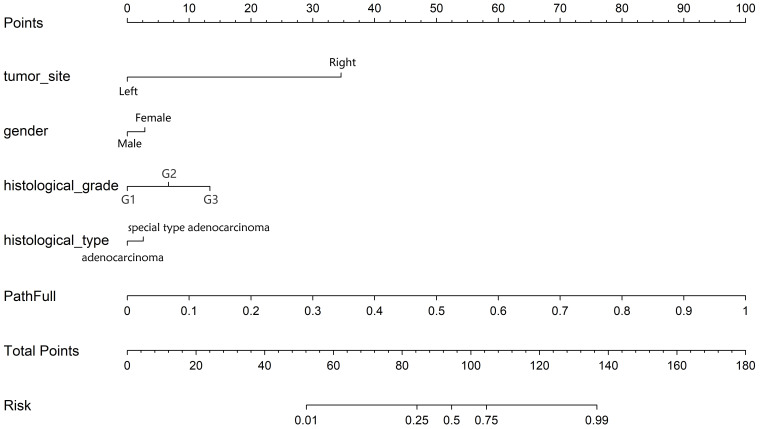
Nomogram prediction model for MSI status.

***Decision curve analysis*** *(* ***DCA)***: **Figure 8** displays the DCA curves for the training, validation, and testing sets. The outcomes highlight the significant advantages of our fusion model in terms of predicted probabilities. In comparison to other models, our fusion model exhibited a greater potential for achieving net benefit. Furthermore, a nomogram was created to improve clinical applicability, as depicted in **Figure 9**.

### Biologic functions associated with MSI status

**Figure 10:**
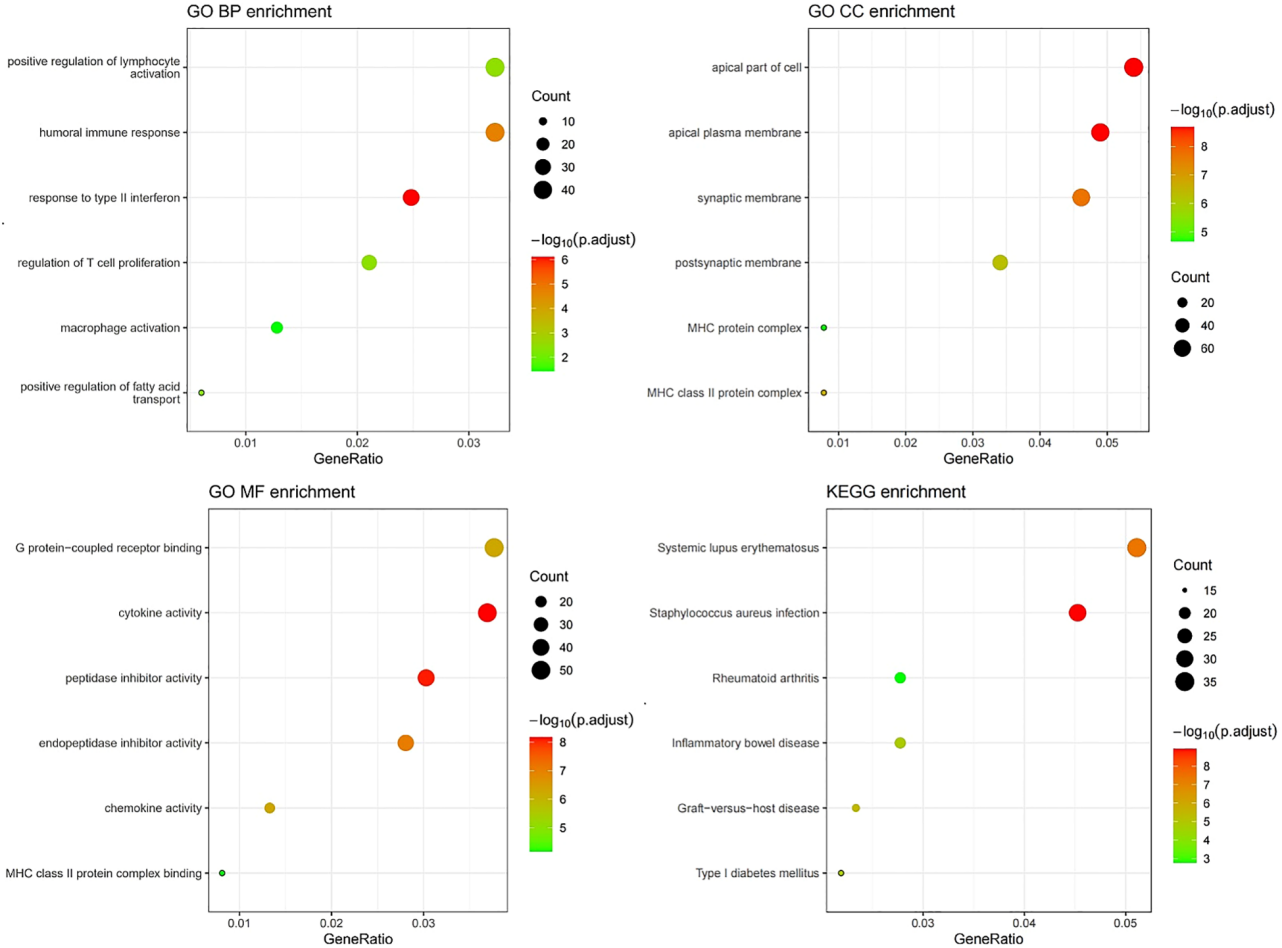
GO/KEGG enrichment analysis correlated with MSI status using RNA sequencing data from the TCGA dataset.

**Figure 11:**
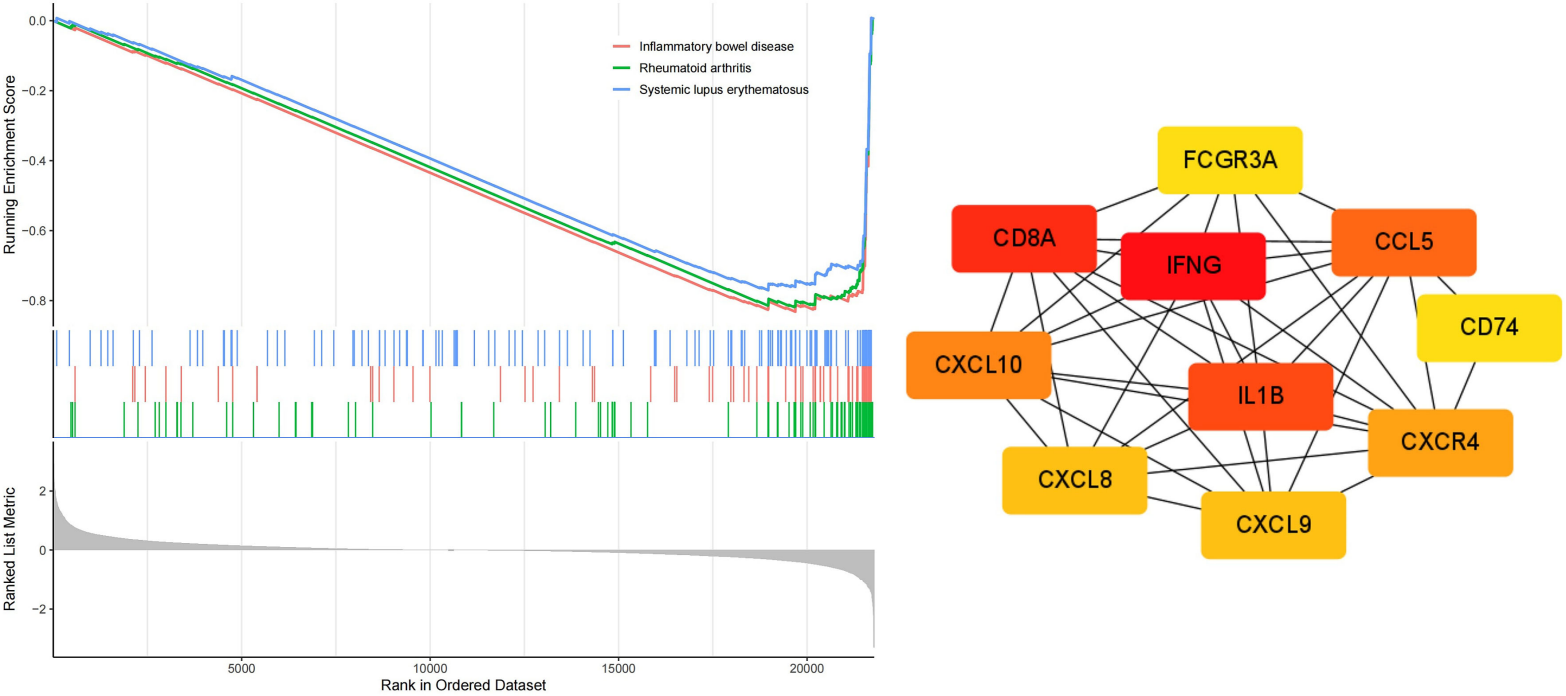
GSEA enrichment and PPI network analysis correlated with MSI status based on RNA sequencing data from the TCGA dataSet.

GO analysis results indicated that the DEGs were primarily enriched in biological processes (BP) related to humoral immune response and regulation of lymphocyte activation, cellular components (CC) such as the apical plasma membrane and MHC protein, as well as molecular functions (MF) including cytokine activity and peptidase inhibitor activity (**Figure 10**). KEGG and GSEA pathway analyses revealed that these genes were predominantly enriched in signaling pathways associated with rheumatoid arthritis, inflammatory bowel disease, and systemic lupus erythematosus(**Figures 10**, **11**). Upon examining a predicted PPI network, the top 10 hub genes, including IFNG, CD8A, IL1B, and CCL5, were identified. These genes are pivotal within the network (**Figure 11**).

## Discussion

MSI is a tumor molecular phenotype resulting from the loss of function in MMR proteins due to deleterious germline mutations, epigenetic inactivation, or somatic biallelic mutations (21). Seminal studies such as Keynote177 have demonstrated that, compared to chemotherapy, Immune Checkpoint Inhibitors (ICIs) can lead to better outcomes in patients with dMMR/MSI-H CRC and that this molecular subtype is closely linked to prognosis (22–24). Recent research has expanded the scope of MSI testing to include treatment decision-making and prognosis across various cancer types (25–27). Although universal screening of CRC patients for MSI status is now recommended, it presents challenges, such as increased workload for pathologists, delays in therapeutic decisions, significant cost increases, and the inability to perform testing in the absence of tissue samples (10). DL offers the potential to streamline MSI testing and expedite decision-making by oncologists in clinical practice. A hybrid model that can be used in clinical practice to predict MSI status was proposed in this study. The primary objectives of our study were: (a) to assess the differences in predictive performance between semi-supervised and fully-supervised DL methods using pathological images; (b) to build and verify a hybrid model to predict MSI based on pathological images and clinical features; and (c) to conduct a pilot study to identify MSI-associated differentially expressed genes.

Given the critical importance of MSI, researchers have explored the use of DL models to predict MSI status from pathological images. Cao et al. demonstrated that a pathomics-based DL model could effectively predict MSI from histopathological images, indicating its generalizability to new patient cohorts (9). Schrammen et al. proposed a slide-level assessment model that uses a single neural network to detect tumors and predict genetic changes directly from standard pathology slides, with AUC of 0.909 for predicting MSI. This approach reduces labor costs by automating the exclusion of normal and uninformative tissue regions (28). Subsequently, Chang et al. developed a method that integrated the CNN model INSIGHT with the self-attention model WiseMSI to predict MSI in CRC (17). Despite achieving an AUC of approximately 0.95 through extensive training with a large sample size, the model did not incorporate the individual clinical characteristics of the patients.

Despite the application of various DL techniques to predict MSI status, which has led to continuous enhancements in the AUC, no research has yet compared semi-supervised and fully-supervised DL methodologies. Consequently, this study conducted a comparative analysis of different DL approaches for pathological omics, and subsequently integrated clinical-specific omics to develop a hybrid model. The findings suggest that fully-supervised pathological models are more effective in predicting MSI, hinting at a potential correlation between MSI status and specific tumor tissue characteristics. Although the study utilized a small sample size, it achieved an AUC of nearly 0.95 in the internal validation cohort and 0.86 in the external test cohort. Recently, French researchers have developed MSIntit, a clinically approved pre-screening tool based on AI for detecting MSI from slides stained with H&E, with a sensitivity of 0.96–0.98 and a specificity of 0.47–0.46 (10). This tool could serve as an optimal screening method, potentially excluding nearly half of the non-MSI-H population and reducing clinical expenses. However, its clinical utility is somewhat restricted due to its non-diagnostic nature.

MSI-H CRC presents with distinct clinical characteristics. Gelsomino F reported a correlation between MSI-H CRC and proximal location, predominantly early stage diagnosis (particularly stage II), poor differentiation, mucinous histology, and BRAF mutations (29). Nakayama concluded that patients with sporadic MSI-H are older, have right-sided colon tumors that are poorly differentiated or mucinous, and exhibit worse overall survival compared to those with Lynch Syndrome (30). Our findings indicated that MSI-H is common among women with poorly differentiated adenocarcinoma in the right colon, particularly those with special types, such as mucinous adenocarcinoma, which corroborated the previous findings.

Furthermore, MSI-related DEGs were commonly associated with signaling pathways implicated in rheumatoid arthritis, inflammatory bowel disease, and systemic lupus erythematosus. Treatment with ICIs has been effective in autoimmune vasculitis, necessitating further investigation into the underlying mechanisms (31). Subsequently, 10 hub genes were identified, including IFNG, CD8A, IL1B, and CCL5. The expression of IFNG, a marker of effector function, is increased in MSI-H gastric cancer than in MSS gastric cancer (32). IL1B, a gene involved in the COX-2/PGE2 pathway, is associated with immune-related adverse events (irAEs) following immune checkpoint blockade (33). These insights provide new directions for immunotherapy and the management of irAEs in CRC.

This study encompassed two cohorts with an acceptable sample size; however, it lacked validation data from multiple centers with larger sample sizes. Consequently, further optimization using multicenter datasets with larger sample sizes across all stages is essential to enhance accuracy and generalizability. Moreover, the exploration of differential genes in this study did not include subsequent validation experiments (such as IHC or knockdown). Future research is needed to further confirm their value.

## Conclusion

In summary, a fully-supervised pathological model outperformed a semi-supervised pathological model in predicting MSI. Furthermore, a hybrid model was developed that employs deep learning algorithms to integrate pathological features with clinical data. This model exhibits exceptionally strong predictive capabilities by leveraging the complementary strengths of both data types to enhance overall accuracy.

## Data Availability

The raw data supporting the conclusions of this article will be made available by the authors, without undue reservation.
