# Supplementary material for: Hybrid model for predicting microsatellite instability in colorectal cancer using hematoxylin & eosin-stained images and clinical features
Source: Front Oncol. 2025 Jun 23;15:1580195. doi: 10.3389/fonc.2025.1580195 (PMC12229802; doi:10.3389/fonc.2025.1580195)
Supplement: Supplementary file 1 [file DataSheet1.docx]

## Supplementary

### 1A. Patch Level Model Training Details

To extend the model's applicability across diverse patient cohorts with significant heterogeneity, we employed transfer learning. This involved initializing the model with pre-trained weights from the ImageNet dataset, which improved the model's adaptability to varied data. A key element of our approach was the careful adjustment of the learning rate to enhance generalization. To achieve this, we utilized the cosine decay learning rate algorithm, as outlined below:

$$\eta_{t}=\eta_{min}^{i}+\frac{1}{2}\left( \eta_{max}^{i}-\eta_{min}^{i} \right)\left( 1+cos\left( \frac{T_{cur}}{T_{i}}\pi\right) \right)$$

In this configuration, the notation $\eta_{\min}^{i}=0$ specifies the minimum learning rate, and $\eta_{\max}^{i}=0.01$ indicates the maximum learning rate. Additionally, $T_{i}=30$ represents the number of iteration epochs in the training process. Other key hyperparameter settings include the use of SGD (Stochastic Gradient Descent) as the optimizer, and softmax cross entropy as the loss function.

### 1B. Multi-Instance Learning-Based Feature Fusion

In this study, we adopted a multi-instance learning-based approach for feature fusion to improve the predictive accuracy of our models. This method involves integrating multiple data points or instances from a single sample to create a comprehensive feature set. Such an approach is essential for effectively analyzing and predicting complex clinical outcomes. Below, we detail the specific steps and techniques employed in this feature fusion process:

1. ***Patch Prediction:*** We utilized the Resnet18 model to predict each patch, obtaining corresponding probabilities and labels, denoted as $Patch_{prob}$ and $Patch_{pred}$, respectively. The prediction probabilities were retained to one decimal places.
2. ***Multi Instance Learning Feature Aggregation:***

- Histogram Feature Aggregation:

1. We treated each distinct number as a "bin" and counted the occurrence of each type of data across these bins.
2. The frequencies of $Patch_{prob}$ and $Patch_{pred}$ falling into each bin were tallied.
3. All features underwent min-max normalization.
4. This process resulted in the generation of $\mathrm{Hist}o_{\mathrm{prob}}$ and $\mathrm{Hist}o_{\mathrm{pred}}$.

- Bag of Words (BoW) Feature Aggregation:

1. Initially, a dictionary was created by identifying unique elements within $Patch_{prob}$ and $Patch_{pred}$.
2. Each patch was then represented as a vector, where the frequency of each dictionary element in the patch was noted.
3. We applied Term Frequency-Inverse Document Frequency (TF-IDF) transformation to these vectors, emphasizing the importance of less frequent but more informative features.
4. This resulted in a BoW feature representation for each patch, encapsulating both the presence and significance of features within a patch.
5. The final BoW features, denoted as $\mathrm{Bo}W_{\mathrm{prob}}$ and $\mathrm{Bo}W_{\mathrm{pred}}$, offered a comprehensive and weighted representation of the patches, suitable for subsequent analytical processes.
6. ***Feature Early Fusion:*** The final stage in our multi-instance learning-based feature fusion involves the integration of the previously derived features: $\mathrm{Hist}o_{\mathrm{prob}}$, $\mathrm{Hist}o_{\mathrm{pred}}$, $\mathrm{Bo}w_{\mathrm{prob}}$, and $\mathrm{Bo}w_{\mathrm{pred}}$. To achieve this, we employ a feature concatenation method, symbolized by $\oplus$, which combines these individual feature sets into a single, comprehensive feature vector. The specific formula for this concatenation is as follows:

$$\mathrm{featur}e_{\mathrm{fusion}}=Histo_{\mathrm{prob}}\oplus\mathrm{Hist}o_{\mathrm{pred}}\oplus\mathrm{Bo}w_{\mathrm{prob}}\oplus\mathrm{Bo}w_{\mathrm{pred}}$$

**1C. Supplementary Table 1. Metrics for Training, Validation, and Test Cohorts in MSI Prediction at the Patch Level Using Semi-Supervised Methods.**

**1D. SupplementaryTable 2. Metrics for the Training, Validation, and Test Cohorts in MSI Prediction Using the Semi-Supervised Pathomics Model.**

**1E. Supplementary Table 3. Metrics for the Training, Validation, and Test Cohorts in MSI Prediction Using the Semi-Supervised Pathomics Model.**

*Supplementary Table 1. Metrics for Training, Validation, and Test Cohorts in MSI Prediction at the Patch Level Using Semi-Supervised Methods.*

| ModelName | Cohort | Acc | AUC | 95% CI | Sensitivity | Specificity | PPV | NPV |
| --- | --- | --- | --- | --- | --- | --- | --- | --- |
| resnet18 | train | 0.852 | 0.937 | 0.9370-0.9376 | 0.850 | 0.852 | 0.554 | 0.963 |
| resnet18 | val | 0.665 | 0.721 | 0.7191-0.7223 | 0.645 | 0.668 | 0.172 | 0.946 |
| resnet18 | test | 0.610 | 0.560 | 0.5584-0.5610 | 0.454 | 0.639 | 0.193 | 0.861 |
| resnet50 | train | 0.893 | 0.965 | 0.9651-0.9655 | 0.890 | 0.894 | 0.645 | 0.974 |
| resnet50 | val | 0.599 | 0.656 | 0.6546-0.6578 | 0.626 | 0.596 | 0.142 | 0.937 |
| resnet50 | test | 0.559 | 0.606 | 0.6048-0.6072 | 0.626 | 0.546 | 0.207 | 0.885 |
| densenet121 | train | 0.886 | 0.959 | 0.9589-0.9594 | 0.875 | 0.888 | 0.629 | 0.970 |
| densenet121 | val | 0.647 | 0.699 | 0.6971-0.7002 | 0.639 | 0.648 | 0.163 | 0.944 |
| densenet121 | test | 0.689 | 0.569 | 0.5674-0.5698 | 0.361 | 0.751 | 0.215 | 0.861 |

*SupplementaryTable 2. Metrics for the Training, Validation, and Test Cohorts in MSI Prediction Using the Semi-Supervised Pathomics Model.*

| model_name | Cohort | Accuracy | AUC | 95% CI | Sensitivity | Specificity | PPV | NPV |
| --- | --- | --- | --- | --- | --- | --- | --- | --- |
| SVM | train | 0.974 | 0.981 | 0.967 - 0.994 | 0.984 | 0.973 | 0.873 | 0.997 |
| SVM | val | 0.679 | 0.786 | 0.664 - 0.908 | 0.769 | 0.671 | 0.164 | 0.972 |
| SVM | test | 0.528 | 0.737 | 0.626 - 0.847 | 0.889 | 0.467 | 0.222 | 0.961 |
| ExtraTrees | train | 0.997 | 1.000 | 1.000 - 1.000 | 0.984 | 1.000 | 1.000 | 0.997 |
| ExtraTrees | val | 0.863 | 0.755 | 0.610 - 0.899 | 0.462 | 0.897 | 0.273 | 0.952 |
| ExtraTrees | test | 0.748 | 0.721 | 0.623 - 0.820 | 0.389 | 0.810 | 0.259 | 0.885 |

*Supplementary Table 3. Metrics for the Training, Validation, and Test Cohorts in MSI Prediction Using the Clinical Model.*

| model_name | Cohort | Accuracy | AUC | 95% CI | Sensitivity | Specificity | PPV | NPV |
| --- | --- | --- | --- | --- | --- | --- | --- | --- |
| SVM | train | 0.744 | 0.811 | 0.753 - 0.869 | 0.810 | 0.732 | 0.367 | 0.952 |
| SVM | val | 0.690 | 0.806 | 0.668 - 0.945 | 0.769 | 0.684 | 0.169 | 0.972 |
| SVM | test | 0.724 | 0.803 | 0.703 - 0.902 | 0.667 | 0.733 | 0.300 | 0.928 |
| ExtraTrees | train | 0.696 | 0.836 | 0.788 - 0.883 | 0.937 | 0.649 | 0.339 | 0.982 |
| ExtraTrees | val | 0.708 | 0.802 | 0.710 - 0.895 | 0.846 | 0.697 | 0.190 | 0.982 |
| ExtraTrees | test | 0.667 | 0.789 | 0.687 - 0.891 | 0.778 | 0.648 | 0.275 | 0.944 |
